# A Tale of Two Perils

**DOI:** 10.1371/journal.pbio.1001503

**Published:** 2013-03-12

**Authors:** Alan S. Kolok

**Affiliations:** Nebraska Watershed Network, Department of Biology, University of Nebraska at Omaha, Omaha, Nebraska, United States of America

## Abstract

Alan Kokol reviews *Peril in the Ponds*, a government scientist's struggle to solve an epidemic of frog deformities in the American Midwest.

[Fig pbio-1001503-g001]In the field of popular science books, science is often presented in an ivory-tower fashion: science strides boldly forward while all other human pursuits, from personal to familial to professional, take a backseat. If only it were so! In reality, the scientific pursuit is, at best, a juggling act where the pursuit of knowledge has to compete with other, sometimes mundane and frustrating, personal and professional responsibilities.

**Figure pbio-1001503-g001:**
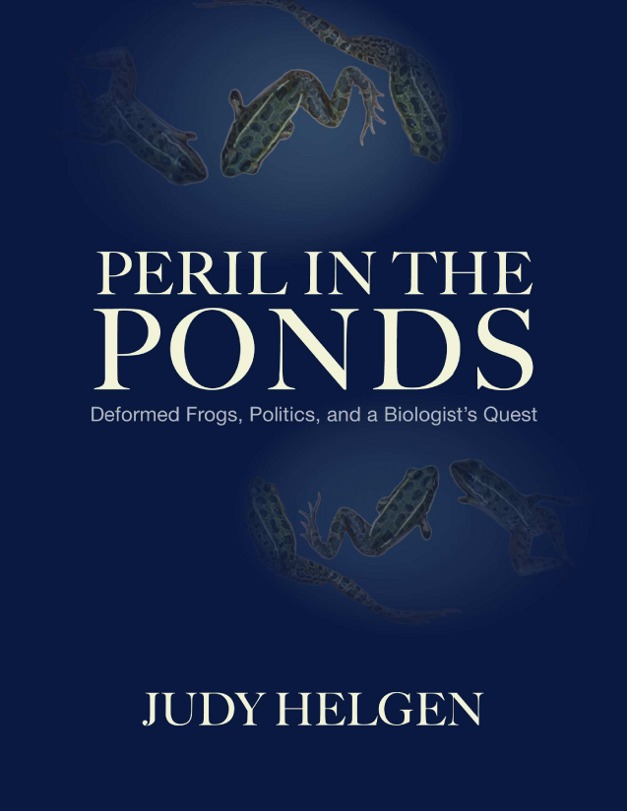
Helgen J (2012) Peril in the Ponds: Deformed Frogs, Politics, and a Biologist's Quest. **Amherst, MA: University of Massachusetts Press. 258 p. ISBN 978-1558499454 (hardcover). US$80.00.**

Judging from its title, you might think Dr. Judy Helgen's book *Peril in the Ponds* belongs in the same genre as Rachel Carson's *Silent Spring*, in that it sounds the alarm about gathering environmental threats to amphibians. While she does focus on the science behind the graphic horror of frog deformities, the book resonates more strongly when it focuses on the personal and immediate struggles of a professional scientist.

*Peril* does a fine job of combining the thrill of a scientific whodunit (just exactly what is responsible for frog deformities in Minnesota?) with a “this-is-my-story” monologue from a professional scientist. And it is the side story of Dr. Helgen's life, interwoven with the realities of modern science, rather than the frog deformities themselves, that steal the show in this book.

*Peril* follows a storyline that begins in 1995, immediately prior to the time when frog deformities become a newsworthy item and grab the attention of the nation. Dr. Helgen focuses on the next few field seasons and on the conflicts that arise while she tries to sustain a field-based program to assess the extent and causative agent underlying the frog deformities.

Amphibians in general and frogs specifically are the only group of vertebrates that undergo a pronounced metamorphosis after they hatch. Anuran amphibians begin life as fishlike tadpoles, then undergo pronounced cellular differentiation and apoptosis to both absorb, in a controlled manner, tissues that are no longer needed (such as the tail) and to express genes and proteins that will generate hind- and forelimbs. Neural and hormonal cell signals are responsible for this metamorphosis, and it appears that such a profound reorganization is highly susceptible to a variety of perturbations, including exposure to chemicals, parasites, excess radiation, and other agents. The story touches upon a number of these potential causes, and systematically rejects each one before moving on to agrichemicals. The difficulties of working backwards from such overt deformities to the mechanistic cause, as this book details, are daunting.

But as compelling as the mystery of the frog deformities may be, this book is also about a passionate scientist observing something that is wrong, perhaps horribly so, and trying to work, against many odds, to study it. It is about lunch-pail scientists who try to conduct serious science while they maintain a family, pay the bills, tangle with administrators who see the agency's priorities in a different light, deal with other scientists' egos, survive the swat-team assault of the media, and search, sometimes vainly, for funding and competent research staff. The book is really a stark and personal account of the realities of modern science, of working in the trenches where scientists butt heads with administrators and fight over scientific decisions every day.

At the end of the day, against such odds, all that anyone can do is to try to uphold the integrity of the scientific process: employ Occam's razor, be systematic, design appropriate experiments, use the proper controls, and so on. The book provides example after example in which administrators, the media, and even other scientists nefariously attempt to manipulate the scientific process for goals that have little to do with science.

Even Dr. Helgen falls prey to these traps. For example, she talks about a study in which eggs from the stock laboratory frog, *Xenopus laevis*, are exposed to water from Minnesota ponds, revealing developmental deformities. Subsequent experiments use bottled water as a negative control to compare to the abnormalities seen in the *Xenopos* embryonic assays where pond water was used. The animals in the negative control also exhibit developmental deformities, prompting Dr. Helgen to speculate that chemicals leaching from the plastic water bottles were causing the effects. While that speculation may ultimately be correct, the scientific logic behind the speculation is flawed, as no experiments are discussed that specifically test this hypothesis. The failure of a negative control to perform as such is a problem inherent in the design of the experiment, not a significant finding in itself.

*Peril* is liberally sprinkled with the word “horror” as a descriptor for the frog deformities, and appropriately so, but why is deformity so much more horrific than outright death? A frog with no hind legs is just a case of metamorphosis gone terribly wrong, and the animal has no future beyond fatal starvation. Would the story have been as newsworthy or as dramatic if the pond was full of dead frogs rather than deformed ones? Is grotesque deformity the charismatic manifestation of pollution that can galvanize political action, in the same way that charismatic megafauna such as polar bears or big cats can galvanize political action focused on conservation?

*Peril in the Ponds* is a worthwhile read and can play an important role as a supplemental text for an environmental science class. It is an excellent segue into conversations regarding the scientific method and how it interdigitates with agency administration, the media, the public, and with policy decisions. It provides an avenue for discussion regarding experimental design, the true nature of science, and the role that scientific discovery plays in society. It also serves as an opening for conversations focusing on career choices and whether the benefits of a scientific career, such as that of Dr. Helgen, are worth the personal costs and professional frustrations.

**About the Author**

Alan Kolok is an environmental toxicologist and director of the Nebraska Watershed Network at the University of Nebraska at Omaha. His research is focused on the biological response of sentinel organisms to toxic compounds, focusing on agrichemicals. He also manages two field stations in Nebraska that conduct research and community outreach. Alan earned his PhD from the University of Colorado in environmental, population, and organismic biology.

